# Acceptability of a family-centered newborn care model among providers and receivers of care in a Public Health Setting: a qualitative study from India

**DOI:** 10.1186/s12913-019-4017-1

**Published:** 2019-03-21

**Authors:** Enisha Sarin, Arti Maria

**Affiliations:** 1 Gurgaon, India; 2 Department of Neonatology, PGIMER & assoc. Dr. RML Hospital, New Delhi, India

**Keywords:** Neonatal health, Family-centered care, Neonatal intensive care unit, India

## Abstract

**Background:**

Family-centered care (FCC), based on collaborative participation of the family along with a team of health care providers, is found to increase the well-being of sick infants in neonatal critical care units. Over the last 4 years, the neonatal unit of Dr. Ram Manohar Lohia Hospital in Delhi has innovated and developed an implementation framework for FCC. This qualitative study assessed the acceptability of family-centered care among providers and family members of neonates to identify gaps and challenges in implementation.

**Methods:**

In-depth interviews were conducted among a purposive sample of twelve family members of admitted neonates and six providers to examine their perceptions and experiences regarding FCC.

**Results:**

Family members and providers expressed a positive perception and acceptance of FCC based on the competencies and knowledge acquired by parents and other caregivers of essential newborn care. Family members reported being satisfied with the overall health care experience due to the transparency of care and allowing them to be by their baby’s bedside. Limitations in the infrastructure or lack of facilities at the public hospital did not seem to dilute these positive perceptions. Providers also perceived FCC as a good practice to be continued in spite of concerns around sharing of nursery space with parents, the need for constant vigilance of parents’ practices in handling of their newborns, and the need for separate, designated nursing staff for FCC.

**Conclusion:**

Both providers and receivers of neonatal care found FCC to be an acceptable form of care. Providers identified challenges and suggested possible solutions, such as need of periodic provider sensitization on FCC, improved staff organization, and provision of mother-friendly facilities to enable her to provide around-the-clock care by her baby’s bedside. Overcoming these challenges would allow for better integration of FCC within general clinical care in neonatal care units.

**Electronic supplementary material:**

The online version of this article (10.1186/s12913-019-4017-1) contains supplementary material, which is available to authorized users.

## Key message

The positive perceptions regarding FCC among stakeholders suggests that scaling up this practice would be beneficial. Evidence of increased competence in neonatal care among parents, improved staff-patient relationships, and positive health benefits for neonates makes a case for further studies in other contexts to make FCC a standard of care practice.

## Background

Family-centered care (FCC) is a philosophy of care based on partnership between the family and the health care team in delivering care to a sick neonate. The collaborative partnership is based on dignity and respect, information sharing, and the family’s participation through their acquired competencies in providing essential newborn care [1]. Parents often report distress, frustration, and alienation if they are excluded from taking care of sick neonates. However, if they are provided with the opportunity to be involved in care, receive clear communication about their newborn’s status from health care providers, and establish rapport with providers, they experience satisfaction and reduced stress [2]. Implementation of FCC has been shown to decrease the length of stay in the hospital for pre-term babies, improve their well-being, allow better allocation of human resources, and enhance parent-infant bonding [3–6].

Specific FCC practices, like kangaroo (skin-to-skin) care provided by parents, improve rates of successful lactation, reduce infant mortality and infection, and increase weight gain [7, 8]. Kangraoo mother care (KMC), additionally, affects brain development of the newborn by stabilizing heart rate, oxygenation, and improving sleep [9]. It is found to have long term effects on IQ and attention among newborns born with neurological vulnerability [10] Among other benefits, KMC reduces a baby’s stress and pain [11] while it may also have maternal benefits such as preventing post partum depression [12]. Mothers who provided KMC were found to have less anxiety and depressive symptoms and more positive interactions with their infants in the first 6 months [13, 14]. Infant massage increases alertness before and after feeding [15], and has been shown to contribute to daily weight gain and reduced length of stay [16]. Massaging infants is also seen to reduce maternal anxiety and depression [17] Breastfeeding becomes possible and frequent when mothers are present in the NICU, which in turn contributes to positive infant health outcomes. Breastfeeding is found to protect against child infections and malocclusion, increases in intelligence, and reductions in overweight and diabetes [18] The mothers who breastfeed are less likely to develop diabetes and to experience breast or ovarian cancer [18] A randomized controlled trial (RCT) in India found that breastfeeding increased among mothers who were involved in FCC for their sick newborns compared to those in the control group whose sick newborns received standard clinical care [19].

Although FCC mainly developed in high-income countries [20], its benefits with respect to infant well-being are increasingly being recognized in developing countries. The practical application of FCC requires a mutual dependence between the family and the health care system. Parents are dependent on the knowledge and expertise of the professionals, and the latter are dependent on the parent’s emotional and physical attachment to the newborn. Thus, the collaboration or partnership is predicated on shared responsibility for the child [21]. Therefore, to evaluate the effectiveness of FCC as a model of care, we must measure and assess not only clinical outcomes but also the working relationship between parents and providers. Indeed, researchers have pointed out the importance of capturing the complicated social relations present in an FCC model, which are affected by attitudes and behaviors of family members and nurses [22]. To date, most of the research on the experience of parents and health care providers in FCC has been conducted in high-income countries. Therefore, the present report, examining the perception and experience of FCC from the perspective of Indian parents and providers, is very timely to help us better understand the application of this model in a developing country setting and its successes and challenges, to move the development of this model forward.

### Setting & infrastructure

Dr. Ram Manohar Lohia (RML) Hospital is a public tertiary hospital in Delhi, India, with a 14-bedded NICU facility catering to patients from Delhi and adjoining states. At any given time, two or three nurses and two resident doctors are available in the NICU. In 2012, RML Hospital began to develop the concept of FCC for implementation in their NICU, where it has now become a standard of care. It was introduced, initially, by the pediatrics team as an informal way of obtaining help from parents in taking care of the newborns and later formalized with the support of hospital authorities. The NICU cares for preterm and sick babies 0–28 days of life at admission with birth weight ranging from 600 g to 3.0 kg and gestational age ranging from 24 weeks to 40 weeks. All infants are outborn transferred to the NICU within 4 to 5 days of birth. All are eligible to receive FCC, except those on ventilator or hemodynamic support. Once infants are out of life support, parental involvement gradually increases to include developmentally supportive care. Two parent-attendants are identified for each baby to ensure that responsibility is shared in order to avoid fatigue of taking care of a sick neonate if the responsibility falls completely on one person. Verbal consent of their willingness to participate is sought, and their details (demographic and contact information) are noted in the admission sheet. They can be mother/father or any accompanying member of family who is willing to participate in care of their baby and are available throughout the hospitalization period of the child. However, not both but at least one is required to be at the hospital 24 h/day as sleeping arrangements for two are limited. Recliner chairs have been provided within the NICU, where parent-attendants can comfortably sit at the baby’s bedside or provide skin-to-skin care. A mothers’ room has also been developed in the vicinity, where mothers can rest.

### Description of FCC intervention

After a baby gets admitted, parents are sensitized about FCC through an introductory session face to face with doctor/nurse. They are provided with an orientation on the concept and importance of FCC, the training process required to become a parent-attendant, and their role in care provision. A training area has been set up, with an audio-visual facility that can seat up to 12–15 people to carry out the daily training sessions scheduled for the parent-attendants accompanying the sick babies, so that they are able to safely participate in care-giving activities for their babies. They also set up a skill station, where doctors and nurses give skill demonstrations on neonatal care, such as how to provide skin-to-skin care; clean, nest, and feed the baby; and maintain personal hygiene. Parent-attendants practice these skills using a warmer with a mannequin and other materials. Informational materials are displayed in the area for parents’ education and information. A dedicated breastfeeding and kangaroo mother care (KMC) room exists within the NICU complex, where mothers can express (manually or by a breast pump) and store their milk (in refrigerator).

Trainings of 30–45 min sessions, and demonstrations are then held for the parent-attendants of all the admitted babies on a daily basis. They are trained to partner in care delivery for their sick neonate using four indigenously developed, culturally sensitive audio-visual training tools that are presented during four skill-building sessions. Session 1 teaches parent-attendants about handwashing, NICU entry protocol, and the importance of personal hygiene to reduce the risk of infection for newborns. Session 2 teaches participants about developmentally supportive care and feeding practices (direct breastfeeding, expressing breast milk, and assisted feeding using feeding devices such as a *paladai*^1^). Session 1 and 2 are covered everyday and all parent attendants are required to attend these, at least once. Session 3 details the importance of and methods to provide kangaroo mother care and is eligible for parent attendants of low weight newborns. Session 4 covers essential topics for providing care at home and identifying danger signs, and is provided before discharge. Furthermore, a ‘Training Guide’ and ‘Operational Guidelines’ have been developed to train nurses on FCC. Content of the nurses training include sensitization on FCC, communication skills, familiarization with content of all modules, hands on practice and skill building of mothers. At the time of the study, an independent outside consultant provided the trainings to parent attendants along with a nurse. Mothers who have been there for a long time and have acquired all newborn care skills are also encouraged to demonstrate skills to the newer ones, and thus, there is considerable task sharing with nurses.

## Methods

### Study design

A qualitative study was conducted in the NICU of RML Hospital over the period October 20 to November 30, 2016. In-depth interviews were conducted with parents and grandparents as well as with nurses and doctors in the NICU.

### Study objectives

The study aimed to: 1. Get a better understanding of the acceptability of FCC from providers’ and clients’ perspectives; 2. Explore the integration of providers’ and clients’ activities in the neonatal unit; and 3. Examine continuing care competencies of parents after discharge.

### Sampling and recruitment

A total of 12 family members (5 mothers, 5 fathers, 1 grandfather, 1 grandmother) and 6 service providers (3 doctors and 3 nurses) were interviewed. The number of family members was determined by the principle of data saturation as, in general, 12 to 15 interviews are considered enough to reach saturation. During the process of interview, we found data saturation in certain research areas after 4–5 interviews. Sampling of family members was done in two cycles. Initially, a list of family members of patients from the register of the last 1 month and those who were currently in NICU for at least a month was used to randomly select the sample by drawing lots. Parents of extremely sick neonates (identified by the head pediatrician) were excluded as it was deemed unethical to engage them in interviews. Those selected who had already been discharged were contacted to assess their availability. Families who stayed outside of Delhi and were unable to participate due to distance were ruled out, and a fresh random lot was drawn. As the number of available participants narrowed, we decided to purposively select family members who were in the NICU for at least 2 weeks. Thus, the final sample of family members were randomly drawn and later purposively selected and consisted of two relatives of discharged infants and ten relatives of inpatient infants at the time of data collection. The sample of service providers was purposively selected comprising a mix of senior and junior nurses and doctors with long and short residency at RML Hospital. We included only doctors and nurses and those who had at least 3 months experience in the NICU, while excluding those who recently joined (< 3 months). Prior appointments were made with selected participants and interviews conducted according to their availability in a private room at the hospital.

### Study instruments

In-depth interview guides (one for parents, one for service providers) were developed with the following key components: perception and experience of FCC and views on its operation and the development of knowledge and skills. Interview guides are appended as Additional files 1, 2 and 3. The guides were semi-structured, containing open-ended probing questions; however, the flow and sequencing of the interviews was left to the interviewers. The interview guides were shared with the consultant in charge of the NICU for feedback, revised, and translated into Hindi. Additionally, consent forms were developed for both family members and service providers.

### Ethical review

The study was reviewed and approved by the RML Hospital Institutional Review Board. Consent forms were developed and required the signature of participants.

### Data collection

Three interviewers (two female and one male), including the lead researcher, conducted the interviews. All the interviewers were outside the purview of the FCC programme and therefore, unlikely to carry any bias to the interview process. All have had prior experience in conducting qualitative research and dealing with sensitive issues. The lead researcher conducted a 1 day orientation training in order to familiarize the other two researchers on the instruments and the data collection process. Each interview took place in a private room at the hospital and lasted from 30 to 90 min. Interviews were audio recorded with prior permission of participants. Interviews were preceded by the reading of the consent form and, upon agreement to participate, were initiated. Interviews with family members were conducted mostly in Hindi while interviews with service providers were conducted in English as well as a mix of English and Hindi. The first three interview transcripts were reviewed by the lead researcher for data quality, and identification of areas where more information is needed. This was shared with the other interviewers.

### Data analysis

Recorded interviews were transcribed and translated by an external agency, and transcripts were then stored in ATLAS.ti, a software program used for coding and analyzing qualitative research. Content analysis was done at first by reading and re-reading transcripts and thereafter applying an a priori coding list (Additional file 1) which was prepared based on the research questions. While a code list helps in reducing the data into meaningful units pre-defined by research questions, it is not an exhaustive tool to analyze inductively. New and emerging codes were added as analysis progressed and pertinent themes could be identified. These themes were then compared across cases to answer the specific research questions. The data were coded and analyzed by the lead researcher and thus may carry a bias in interpretation. Efforts were made to minimize the bias by presenting themes, key findings and having discussion with the other researchers on a regular basis.

## Results

As seen in Table 1, four newborns admitted to the NICU were from Delhi while the majority were from the nearby states of Haryana and Uttar Pradesh. The age of parents ranged from 19 years to 34 years; years of education ranged from completion of 5th class up to post-graduation; and the occupation of fathers (the primary breadwinners in all interviewed families) was a mix of private business, daily wage labor, and government employment. Thus, there was wide variation in the income and wealth level of the families. Each parent interviewed corresponded to a unique newborn; the grandmother and grandfather corresponded to the same newborn. The neonates admitted to the NICU were the first child for the majority (*n* = 10) of the parents, and their age at admission ranged from 2 days to 15 days of birth with an average age of 7.4 days. The average gestational age was 27.5 weeks, and their weights ranged from 600 g to 1600 g. (Table 1). All the mothers and four of the fathers interviewed stayed at the hospital. Four of the husbands of interviewed mothers visited the hospital daily after work. The length of stay in the NICU for newborns ranged from 14 days to 106 days. All the neonates were admitted previously to other facilities for an average period of 5 days before being transported to RML Hospital due to complications.

**Table 1:** Demographic characteristics of family members

Participant ID	Residence	Age	Education	Occupation	Birth order of neonate	Gestational age (weeks)	Weight of neonate at time of birth (gm)	Length of stay in NICU (days)
F* 01	Delhi	32	12	Restaurant owner	1 (twins)	28	1300	34
F02	Sonepat, Haryana	34	Graduate^†^	Government officer	1	28	missing	20
F03	Palwal, Haryana	32	12	Shopkeeper	3	24	1100	55
F04	Delhi	26	missing	Contractor	1	28	900	106
F05	Delhi	26	Graduate	Engineer	1	23	600	61
GF*01	Shamli, Uttar Pradesh	62	Graduate	Retired	1 (twins)	30	1600; 1400	18
GM* 01	Shamli, Uttar Pradesh	61	5	Homemaker	1 (twins)	30	1600; 1400	18
M*01	Sonepat, Haryana	22	8	Homemaker (husband is a professional at a private company)	1 (twins)	28	800; 600	55
M02	Faridabad, Haryana	19	10	Homemaker (husband is a farmer)	1 (twins – one died immediately after birth)	28	900	14
M03	Delhi	24	Master of Commerce	Homemaker	1 (twins)	26	1000; 810	37
M04	Faridabad, Haryana	25	10	Homemaker	1	28	1000	37
M05	Meerut, Uttar Pradesh	32	10	Homemaker (husband is a daily wage laborer)	4	32	missing	55

* *F* Father, *M* Mother, *GM* Grandmother, *GF* Grandfather ^†^Completion of undergraduate education

Apart from family members, service providers were also interviewed. Three doctors, each working in this NICU for the last 6 to 8 months, and three nurses were interviewed. The doctors were pediatric residents and were appointed on a rotational basis. Among the nurses, one was a senior nurse (sister in-charge) and the other two staff nurses attached to the NICU.

The qualitative analysis delineated the following major themes. We present the parents’ views first, followed by providers’ views on the same themes.

### Acceptability of FCC among family members was based on gains in knowledge, access to the child, and improved well-being of the child

Family members juxtaposed their experience in the RML NICU to their previous experience in other facilities while talking about their understanding of FCC. They described their experience in previous care units as one of helplessness, where they had little information about their child and were only sporadically informed of the child’s status. They reported that their babies were better cared for after coming to the RML NICU. They felt that doctors were more accessible to them, and they saw doctors frequently checking on their babies. They also reported more polite staff behavior and an increase in knowledge of how to take care of their babies.

“This is good because if we cannot see meet our children, then how would attachment develop and how would our child know us and feel us? How would they know that we are their parents? It is the good part that we are able to stay here and can take care of our babies. We can see what doctors are doing and what is the temperature of child. We are being informed about machines as well now.” (Mother 03).

Interestingly, fathers talked about the difference in expenses between previous facilities, which were mostly private facilities, and the current one and expressed surprise that the latter provided better care, perhaps a result of their perception that free or subsidized health care offered at public institutions cannot be of good quality. The biggest gain, according to parents, has been in acquiring knowledge of essential newborn care. Parents described the components of newborn care and narrated their experience of how they kept their baby clean and warm, and how they kept themselves clean.

### Acceptability of FCC among service providers related to gains in neonate health and potential reduction of blame in case of adverse events

Despite their initial hesitation when the concept was introduced (largely because they were used to working on critical care under strictly isolated conditions), all providers reported that they now have a favorable attitude to FCC after realizing its importance in building attachment between the newborn and his or her caregivers, which leads to positive clinical outcomes. They also perceived FCC as a tool to reduce staff workload and mitigate potential conflicts with relatives of newborns who have an adverse outcome by reducing the likelihood of blame. Service providers, additionally, viewed FCC as an empowering tool for parents, whose caregiving skills were enhanced, which would potentially ensure better continuity of care at home.

*“Initially, mothers used to hesitate, especially if [this was] their first delivery, while handling and taking care of their newborns. But after coming here, they feel confident and are in a better position to nurture their babies at home properly.” (Nurse 06).*

### Family members adapted to the NICU environment despite structural stressors

Family members narrated their daily routine as consisting of cleaning and feeding their baby, providing skin-to-skin care, going out to eat, and not having enough sleep. Mothers did not generally express feeling any stress, besides lack of sleep, except one who reported being stressed about not eating a proper diet and her consequent inability to express breastmilk. A few respondents felt that nurses should share some of their duties at night, such as feeding, suggesting that mothers did not get enough rest. Mothers had to express breastmilk before each feeding, which occurred every 3 hours. Although there was a refrigerator for storing milk, mothers were encouraged to express every time their babies were fed to ensure milk production and to stimulate the suckling reflex of infants. A few mothers felt that feeding infants with stored milk at night would provide them with sufficient rest. Apart from this, parents, in general, did not report much stress, perhaps because most had support from their extended family. Respondents with family in Delhi were provided with food from their relatives’ house. Skin-to-skin care was also provided by visiting relatives along with fathers.

Most fathers who were interviewed had taken leave from their jobs to stay in the hospital with their baby. They did not report concern about their absence from work as for most this was their first newborn, whose well-being was perceived to be more important. Fathers were involved either directly, by staying at the hospital, providing skin-to-skin care, and feeding (if mother is absent), or indirectly, by buying medicines (presumably for the mother and himself) and doing other errands. Most of these fathers fit into traditionally male roles in their families, but when they were part of the FCC, they were encouraged by the nurses to undertake typically nurturing roles, such as feeding and cleaning their infant. All the fathers expressed satisfaction in being able to provide this kind of care to their newborns.

*“Earlier only mothers used to come. Fathers never came because they used to think it is the work of the female/mother. But because of this FCC, when mothers are not able to come, fathers come, they also take an interest, we even force them to do the work by saying that do this, it’s your work also. So, the father also takes an interest and feeds [his infant]. Even when fathers are not able to come, so the baby’s uncle comes and feeds. So, it’s a really good thing. Parents’ involvement is a good thing.” (Nurse 01).*

Respondents reported stressors related to the absence of sleeping arrangements at the hospital and the lack of bathrooms and hand basins in close proximity. Mothers slept indoors in the waiting area on chairs if their children were in intensive care, and those whose children were improving slept in two separate rooms, with their infants, which adjoined the nursery and had two cots each. However, fathers had to sleep outside the NICU in the landing area in front of an elevator; a space big enough to accommodate about 8–10 people, who spread sheets and blankets on the floor at night to sleep. Parents explained that in the absence of rooms, it was difficult to stow away the bedding that they rolled out at night to sleep. Lockers are provided, but they are too small to hold anything besides medicines and diapers.

### Interaction between staff and parent-attendants was based on helpful communication although moderated by inherent hierarchical patterns that differentiate compliant and non-complaint parents

A common theme that emerged in many of the interviews with family members was their gratitude to the hospital staff for behaving well with them, patiently instructing them in their baby’s care, and being attentive to their babies, as reflected in this quote from a grandmother:

“*Behavior is good. They [the staff at the RML Hospital NICU] explain everything nicely about taking care of the baby after going back home and how to maintain hygiene. The doctors are also well behaved. They take care of the babies and patients well.” (Grandmother 01).*

Mother-nurse interaction was reportedly positive. Mothers spoke about how the FCC nurses were different from nursing staff they had seen in other hospitals. Parents were happy with their interactions with the staff, with two of the respondents also justifying the occasional scolding or bad mood of the staff, as this father reports:

“*Yes, the nature of everyone here is quite good. They scold us sometimes, but it doesn’t matter because it is for the betterment of our baby, so it’s okay*.” (Father 02).

Service providers, however, did not share the same benevolent attitude toward the parents. They reported that some mothers did not follow instructions properly, either because they were not educated and did not understand the importance of preventing infection, or they were tired due to lack of sleep and proper diet. Lack of education, poverty, and the birth order of the neonate (parents with previous children being less compliant than those of first born) were perceived to be major factors in non-compliance of parents. Nurses reported that such parents considered FCC as a temporary schedule and neglected their tasks when they thought they were not being watched, as reported by this nurse:

“*There are some mothers who take things very casually. They feel that they are not being watched, so they won’t do it and go away. It’s like they come four times, but wash their hands only once. And, if we see this, we send them back and ask them to wash their hands. So, everything depends on the education level and because they are going to go back to the same society… and what they learn from here is basically I think so depends on… because whatever they have been following for so many years… it cannot be changed in one, two, three, four days.”* (Nurse 02).

### Integration of the FCC program in the NICU: parents adjust within the hospital environment while service providers adapt to parental involvement with certain reservations

As parents talked about their day-to-day life at the hospital by giving examples of their activities on a typical day, it is evident that they slowly adjusted to the routine of hospital life, despite its limitations. After an initial period of hesitation and fear, parents gradually fell into the regular rhythm of hospital life. They did not report feeling that FCC was a burden or feeling like an outsider.

Fathers reported that although they adjusted to the routine, their having to be outside and sleep in cramped spaces placed stress on them. As they were not allowed inside the NICU all the time, they took on their regular “provider” role: bringing food for their wife, buying medicines, and doing outside errands. However, as the FCC program at RML Hospital focused on the father’s role in providing skin-to-skin care, most of them took up this role, and, although initially afraid they would drop or harm the baby (echoing other studies [23, 24]), they gradually grew to savor this role.

Two themes emerged from doctors’ and nurses’ views on the integration of FCC in the NICU structure and organization. One is the *identification of FCC duty as separate from regular clinical duties,* under which are the subthemes: *shortage of staff* and *non-compliant parents create more work.* The other major theme is *clinical benefits outweigh disadvantages of FCC.*

#### Identification of FCC duty as additional work

Providers reported that although FCC has become part of the routine, when there was a shortage of nursing staff, they felt their regular work was disturbed, and staff felt overloaded as they then had to perform their clinical duties as well as instruct guardians. Nurses spoke of FCC duties as being separate from their regular clinical work and identified these as *instructing and monitoring* the parents, during which they not only pay attention to details such as how the mother sits during KMC and instructing her on how to hold the baby, but they also make sure that she is not falling asleep, implying a constant vigilance over the parents. One doctor had this to say:

*“I think [FCC] is not integrated completely because we have [a] shortage of staff. Sometimes we do get angry, especially in the morning, because we need a good number of nurses, but in the morning we have three nurses. If we take one, then 30% is gone. So, that is the problem.” (Doctor 02).*

According to another doctor, new parents have to be continuously monitored since they were likely to touch or do things inside the nursery that may be harmful to the health outcomes of babies in the NICU. Thus, monitoring of parents is a responsibility that is perceived to be an “additional” task, yet one which doctors were obliged to undertake given the need to be extremely careful about infection control within the nursery. They also worried about the nursery getting crowded because then it was difficult to monitor parents and ensure infection prevention protocols were being followed.

#### Non-compliant parents create more work

While nurses felt that parents taking over cleaning and feeding babies eased their workload, they also believed that inattentive or negligent parents actually increased their work as then they had to constantly watch over such parents. This nurse expresses her opinion about how well FCC is integrated into overall NICU care:

“*It will take time because we, as caretakers, remain and the mothers keep changing. In case we get good mothers, the one or two months they remain it goes nicely and smoothly. But if we get an irritated mother, we get irritated too. So, it basically depends on the mothers*.” (Nurse 03).

#### Clinical benefits seen to outweigh disadvantages of FCC

Despite the overarching concerns about FCC creating extra work and its possible negative effects on infection control, providers viewed FCC in a positive light and appeared to have accepted it as a standard of care as they see the benefit of parental presence for the well-being of the baby.

### Empowerment and self-efficacy of parents

Parents reported greater capability to take care of their newborns. Mothers, especially, felt empowered to take better care of their babies and expressed confidence about continuing their care at home, even in the face of cultural and familial impediments. The following is a segment of an interview with a mother:

*R: I will do at home what I am doing here.*

*I: But at home you have many people and it is not that everyone will come with their hands washed, right?*

*R: I will tell them to wash, I will tell them to do like this, otherwise he will be sick again… After all this effort, we take him out of the hospital and he will again go to the hospital, so don’t do that. So, I will not allow them to touch [him] without washing [their] hands. (Mother 03).*

## Discussion

Family-centered care, a concept of care that arose predominately in high-income countries, is steadily entering low- and middle-income country settings, and the RML Hospital NICU is one such public health facility in India that is using this model. Notwithstanding the reduced space and staff compared to that available in high-income country facilities, the fact that parents reported a positive experience and perceived FCC as being greatly beneficial to their children is a success in itself. Similarly, service providers see the enormous benefits of FCC to a newborn’s health and therefore have “bought in” to the program despite increased workload and provider concerns about infection prevention.

In keeping with a recent critique of FCC [25–27], while we found a very positive perception of the concept overall, we also identified structural and organizational challenges, including a lack of sleeping arrangements for parents and limitation in nursing staff organization, that need to be addressed if the model is to be sustained and scaled up.

Our study found that parents adapted to the hospital environment and provided essential care to their infants as they were trained. The nurses had clear expectations of what activities parents had to perform, parents followed their instructions, and there was no intention on the part of the nurses to negotiate about how often mothers had to express milk and feed their infants at night although it was an issue the parents were concerned about. Conflict can arise when parents do not comply with nurses’ instructions and expectations [28–30]. We observed how health care providers began to label parents that did not follow instructions, indicating a lack of trust on the part of the providers in these caregivers’ abilities to care for their child in the NICU and at home. Currently, nurses go through a training on FCC prior to being involved in the program. If aspects of interaction and communication with parents were included in the training, it would help in reducing conflict. The critical role of effective communication in ensuring parents feel listened to, respected, and thereby gain confidence has been highlighted in several studies [31–33].

In general, family members reported positive interactions with staff. This was typically in contrast to patients’ experience with and reports of staff behavior in other public health facilities in India. Many patients in maternal care in Indian health facilities report abusive behavior from staff, including scolding and hitting, which the patients themselves normalize and justify citing the workload of staff, limited space, and heavy load of patients [34]. But, as pointed out by our results, there is already a positive relationship between parents and providers in this unit, a noteworthy achievement of the FCC model, which has the potential to promote good practices in patient-provider interactions. This positivity can be leveraged to further improve interactions through regular staff feedback meetings.

Mothers were found to adjust to the hospital environment without getting into power struggle with the nurses, perhaps a result of societal hierarchy reflected in the hospital environment. Mothers’ experiences in a NICU in England were slightly different wherein a theme of “finding my place” was identified, with mothers often feeling alienated in the clinical space of FCC. They experienced power struggles with nurses as they asserted their maternal role but to whom they deferred in order to have an amicable relationship [35].

In keeping with other studies, we also found that involvement in care led to greater maternal confidence in providing care to the infant [36–39]. The way the FCC model is designed at RML Hospital, parents are provided with clear audio-visual information and repeated interactive education, which has built their capacity to perform care activities and, thus, their confidence. Additionally, in the Indian setting, extended family is a source of social capital, which in turn is a factor in increasing maternal well-being and confidence [40], as sharing duties makes a mother less fatigued and hence more able to take care of her baby. Education and training to encourage care in line with hygienic practices is especially relevant to India, where infections are a leading cause of infant and child mortality. Competency in caregiving is therefore expected to have long-term consequences on the child’s health. However, mothers did report the challenge of providing constant care when they were often in poor health themselves (due to recent caesarian section or other birth complication), something that has been raised in other studies [26]. For instance, in this setting, mothers reported the stress of having to wake up every 3 hours to feed their infant. Greater infrastructural support could reduce this burden.

Fathers in our study reported a positive experience. They noted the cost difference between private facilities and the current one, expressing surprise that the latter provided better care, perhaps a result of their perception that free or subsidized health care offered at public institutions cannot be of good quality. They played a prominent role in some aspects of care giving. They were generally positive about their experience, although they complained about the absence of sleeping facilities. In a review of fathers’ role in the NICU, barriers such as “the physical aspect of the infant [small, sick], the environmental characteristics of the NICU environment, and their own feelings and fear about parenting a fragile infant” were reported by fathers [41]. Indian fathers in our study were not very different. They initially feared holding their infant due to his or her small size, but soon overcame this fear and were seen to be engaged in caregiving. In contrast, an Italian study found that fathers felt a loss of control and disengaged from the care process in FCC [41], an experience shared by Iranian fathers in another study, who additionally felt shame for having a premature baby [20]. In our study, however, we did not come across any such feelings from fathers who stayed at the hospital. This could be a result of frequent interaction with nurses, who provided them with the necessary motivation and guidance to perform a few key duties.

In a space where anxious parents and providers meet and interact all the time, there is bound to be a level of frustration among the latter as they focus on the technical care of the newborn. Although a redesign of the unit has created private spaces, there are constraints in terms of in-stay facilities. Several Western studies point to the importance of providing single family rooms in increasing parents’ participation [38, 42–45], decreasing infection, decreasing medical procedures, and having an overall positive effect on a child’s weight. An Indian public health facility has limited resources to provide such amenities. However, a separate space enabling both parents to stay would be ideal. Additional amenities, such as large lockers to store luggage and washrooms in close proximity to sleeping quarters with hand-basins to wash hands after eating, are also needed to make parents’ stay more comfortable and stress-free.

Designation of current staff to FCC is perceived as affecting regular work, which adds to the stress of nurses and doctors. Doctors were not comfortable with presence of parents during ward visits. A global review of the literature assessing FCC programs reveals that providers’ views about parental presence are mixed. Those who prioritized clinical success felt that procedures were complicated with parental presence in wards [25]. Our study had a similar finding. Furthermore, staff felt that FCC was an additional burden on staff time due to the continuous monitoring and instructing of parents. Only once the FCC becomes part and parcel of the NICU functioning and organization, and not viewed as something separate, will it begin to be normalized as regular course of work. A restructuring of staff duties, so that they no longer feel burdened with additional work, can be introduced by the management through a reasonable rotation of staff. Furthermore, ensuring that parents do not crowd the nursery during ward visits of doctors or during procedures would help in reducing doctors’ stress and increasing their trust in the program.

## Limitations

A limitation of our study is that we did not include inter-rater coding and interpretation. Moreover, the conclusions we draw from the study are to be treated with caution as it was conducted in a hospital that has already been working on FCC and therefore, has had additional support structures which may not be available in other hospital settings. Therefore, the model needs to be tested in different settings.

## Conclusion

Both guardians and providers in this Delhi hospital generally found FCC to be an acceptable model of care. The gains observed in improved newborn care skills among parents which would presumably continue back at home are positive steps in neonatal care. Improvement in gaps and challenges identified by the study is expected to lead to a more cohesive and integrated program. In 2017, the Ministry of Health and Family Welfare also came out with operational guidelines for the implementation of FCC in public health facilities in the country and has initially earmarked 700 facilities for implementation [46]. Further studies in these contexts are needed to test FCC as a standard of care.

## Additional files

Additional file 1: Code list (RTF 52 kb)

Additional file 2: Interview guide for service providers. (DOC 25 kb)

Additional file 3: Interview guide for Parent-attendants. (DOC 31 kb)

## Abbreviations

FCC: Family centered care
IDI: In depth interview
KMC: Kangaroo mother care
NICU: Neonatal intensive care unit
NSS: Non- nutritive sucking
RML: Dr. Ram Manohar Lohiya Hospital
SNCU: Special newborn care unit

## Footnotes

1 A *paladai* is a traditional feeding device used in India which allows the caretaker to gently pour expressed breastmilk or other liquids into the infant’s mouth.
