# Supplementary material for: Acceptability of a family-centered newborn care model among providers and receivers of care in a Public Health Setting: a qualitative study from India
Source: BMC Health Serv Res. 2019 Mar 21;19:184. doi: 10.1186/s12913-019-4017-1 (PMC6427855; doi:10.1186/s12913-019-4017-1)
Supplement: Supplementary file 1 — Code list (RTF 52 kb) [file 12913_2019_4017_MOESM1_ESM.rtf]

Appendix 1
Code-Filter: All
______________________________________________________________________

HU:	FCC_RML qual
File:	 [C:\Users\Enisha\Documents\My Docs Backuup-290617\FCC_RML qual.hpr7]
Edited by:	Super
Date/Time:	2019-01-25 09:53:47
______________________________________________________________________

Admission : 		All about admission to RML NICU
Adv_FCC :	 	Advantages of FCC
Comp_non FCC: 		Complaints of non FCC units
Comp_Oth Hosp: 	Complaints of other hospitals
Compliance: 		Compliance to instructions
Disadv_FCC: 		Disadvantages of FCC
Doc role: 		Doctor's role
Effect_Proflife: 		Effects on professional life
extra work: 		Extra work FCC
Fam Sup:		Family Support
Father's role:		Father's role
FCC duty: 		FCC duties/tasks of nurses
Feeding: 		Feeding practices
First Treat: 		First treatment of infant
Follow Up Hosp: 	Follow up at RML hospital after discharge
FU_ENC: 		Follow up with essential newborn care at home 
FU_Unable: 		Inability to follow with newborn care at home
Gender:			Issues/care of newborn girl vs boy related to gender
Hosp Stay:		Experience of stay at RML hospital of parents
Impact_Newborn: 	Effects of FCC on newborn health
Infra: 			Infrastructure support
Integration: 		Integration of FCC into NICU
Know_ENC: 		Knowledge of essential newborn care
New Knowledge: 	Newly acquired knowledge of newborn care for parents
Nurse role: 		Role of nurse in FCC NICU
Obj_FCC: 		Objectives of FCC
Par beh: 		Parents' behavior at NICU
Percep_FCC:		Perception of FCC
Preg history: 		Pregnancy history of mother
Preterm: 		Preterm related text
Prob_Del: 		Problems in delivery of newborn
Prob_Newborn: 		Health problems with newborn 
Prob_Preg: 		Complications in pregnancy
Provision: 		Provision at RML
Reas_shift hosp:		Reason for transferring to RML NICU
Staff Beh: 		Staff behavior
Staff No: 		Staff number
Stress: 			Stress of parents
Stress_BF:		Stress related to breastfeeding
Sugg: 			Suggestions by parents and providers for improvement of FCC NICU
Training: 		Training 
Trans know: 		Transfer of knowledge from nurses to parents
Typ Day: 		Typical day description of parents at FCC NICU
Typ Day_diff: 		Difference in typical day
Typ day_Doc: 		Typical day description of doctors
