# Supplementary material for: Acceptability of a family-centered newborn care model among providers and receivers of care in a Public Health Setting: a qualitative study from India
Source: BMC Health Serv Res. 2019 Mar 21;19:184. doi: 10.1186/s12913-019-4017-1 (PMC6427855; doi:10.1186/s12913-019-4017-1)
Supplement: Supplementary file 2 — Interview guide for service providers. (DOC 25 kb) [file 12913_2019_4017_MOESM2_ESM.doc]

## **In depth Interview Guide**

**Checklist for Demographic Data**

Date of interview : ____/ ____/ ____ (dd/mm/yyyy)

Unique identification number (UDI):

Type of study participant:

Provider__________________Client____________

Category of client_____________

Language:

Location:

Time started : __ __

Time ended: __ __

Name of interviewer  : _________________________________________________________

Age of respondent : _______________ (in completed years)

**IDI Questions**

1. Can you tell me a little about yourself?

Probes:

(How many years have you been working here? What’s your degree? Where are you from?)

1. Can you tell me something about Family Centered Care?
2. (What is it about? Objectives? How is it different from other SNCU which does not have FCC? Why it was implemented etc)
3. What are your views on FCC?

Probes:
How do you feel this kind of care affects a newborn? Describe. Give examples.

How do you feel about your involvement? Does it stress you? Does it make you feel useful? Why? Why not?

1. How has your experience been working in FCC?

Probes:

Tell me about a typical day you spend here? (what exactly do you do during your working hours?)

How do you feel FCC has affected your clinical skills and knowledge?

How do you feel when you have to teach and counsel mothers?

1. What do you think are the greatest advantages of FCC?
2. Now can you tell me if there are any disadvantages? If so, please explain.
3. Would you like to suggest anything to improve the programme?
