# Supplementary material for: Acceptability of a family-centered newborn care model among providers and receivers of care in a Public Health Setting: a qualitative study from India
Source: BMC Health Serv Res. 2019 Mar 21;19:184. doi: 10.1186/s12913-019-4017-1 (PMC6427855; doi:10.1186/s12913-019-4017-1)
Supplement: Supplementary file 3 — Interview guide for Parent-attendants. (DOC 31 kb) [file 12913_2019_4017_MOESM3_ESM.doc]

## **In depth Interview Guide**

***Instructions***

The suggested questions provided in the guides below allow for in-depth and comprehensive interviews of participants. The questions are set to elicit their experiences and perceptions of why and how their attitude toward contraceptive use changed. Wherever necessary, the interviewer must ask about examples and experiences from the participant’s life.

It is important to note that this type of interview is not an oral questionnaire. In no way should the interviewer use this guide as a survey tool but must use the questions as triggers to a deeper understanding. The open-ended questions allow for a maximum expansion of the answers as well as for additional questions which allow the interviewer to probe related issues which may arise during any stage of the in-depth interview. Useful probes include why, when, how, how often, can you tell me more about.., etc.

The interviewer should take comprehensive notes during the interview (even if a tape recorder is being used). The interviewer’s impressions of the interview process should be put in writing as soon as the interview is over, including the difficulties that may have arisen, the participant’s level of collaboration, as well as the interviewer’s observations, comments and feelings.

After the introduction, the basic demographic data indicated in the following check-list, should be collected for analysis purposes:

**Checklist for Demographic Data**

Date of interview : ____/ ____/ ____ (dd/mm/yyyy)

Unique identification number (UDI):

Type of study participant:

Provider__________________Client____________

Category of client_____________

Language:

Location:

Time started : __ __

Time ended: __ __

Name of interviewer  : _________________________________________________________

Age of respondent : _______________ (in completed years)

**The following questions need to be asked of clients**

birth order of neonate:_______________

number of children: ____________________

Level of education:_______________________

Occupation: ______________________________

**IDI Questions**

1. Can you tell me a little about yourself?

Probes:

(Where you are from, what do you do, how long have you been married, how many children you have, who is there at home, how did your baby land up here, how was your pregnancy, what are your thoughts about why your baby is sick etc..)

1. What do you know of family centered care? (an explanation may needed to be provided, such as: This system of taking care of your baby while being at the hospital..)

Probes:

Can you tell me what is the basic objective of family centred care?

What have you learnt about this kind of care? Please describe

What are the things you did not know before that you have learnt here? Please elaborate.

1. What do you feel about this system of FCC?

Probes:
How do you feel this kind of care will affect your child? Describe

How do you feel about your involvement? Does it stress you? Does it make you feel useful? Why? Why not?

How do you feel about what is happening back home?

What do you most like about this system of care? Describe

What don’t you like about this system of care? Describe

**For those who are currently in the hospital**

1. Do you feel you will be capable of caring for your child at home? Why do you feel that way/or Why don’t you feel that way?
2. Tell me about a typical day you spend here? (right since the time s/he wakes up until bed time at night. If respondent cannot recall, ask about yesterday)
3. Can you tell me what happens when the day is not typical, such as when the baby has taken a turn for the worse…Describe
4. How does your family support you while you take care of your child? Describe
5. Can you tell me about your breastfeeding regimen? (Probe about ability to breastfeed, amount, biggest worries…)
6. Do you feel you can do all the things advised by the doctor or nurse? Why? Why not?
7. Would you like to suggest anything to make yourself more capable of looking a

after your baby?

1. Would you like to suggest anything that will make your stay more comfortable?

**For those who were discharged**

1. Can you tell me a little about yourself?

Probes:

(Where you are from, what do you do, how long have you been married, how many children you have, who is there at home, how did your baby land up here, how was your pregnancy, what are your thoughts about why your baby is sick etc..)

1. What do you know of family centered care? (an explanation may needed to be provided, such as: This system of taking care of your baby while being at the hospital..)

Probes:

Can you tell me what is the basic objective of family centred care?

What have you learnt about this kind of care? Please describe

What are the things you did not know before that you have learnt here? Please elaborate.

1. What do you feel about this system of FCC?

Probes:
How did you feel this kind of care affected your child? Describe

How did you feel about your involvement? Did it stress you? Did it make you feel useful? Why? Why not?

How did you feel about what was happening back home?

What did you most like about this system of care? Describe

What didn’t you like about this system of care? Describe

4. How did you use what you learnt at the hospital to take care of your baby after you came back? Ask for examples

5. Did your baby fell ill after discharge from the hospital? How many times? If yes, what did you do…describe…give example. (Interviewers please find if any learnings from FCC)

6. What are you feeding your baby? (If respondent answers that she is giving other than breastmilk, explore reasons why…)

7. Can you tell about a typical day you spent at the nursery when your child was admitted?.....describe

8. How did you family support you while you were there and now while you are back at home?

9. Do you feel you can do all the things advised by the doctor or nurse? Why? Why not?

10. Would you like to suggest anything to make yourself more capable of looking after your baby?

11. Can you suggest anything that would have made your stay more comfortable in the hospital?
